# Supplementary material for: Autophagy acts through TRAF3 and RELB to regulate gene expression via antagonism of SMAD proteins
Source: Nat Commun. 2017 Nov 16;8:1537. doi: 10.1038/s41467-017-00859-z (PMC5691083; doi:10.1038/s41467-017-00859-z)
Supplement: Supplementary file 1 — Supplementary Information [file 41467_2017_859_MOESM1_ESM.pdf]

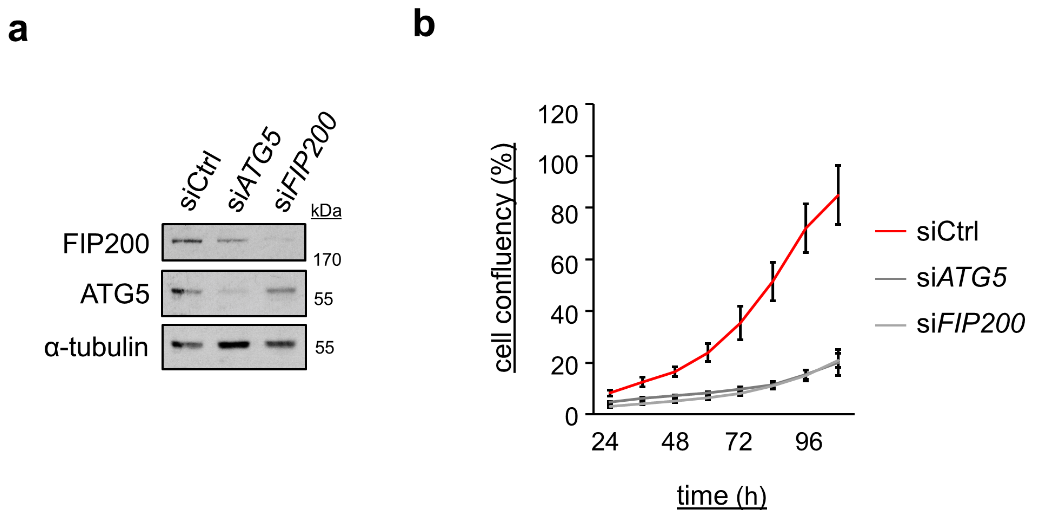

Supplementary Figure 1

a) A549 cells were transfected with non-targeting control siRNA (siCtrl) or siRNA (si) targeting the indicated transcripts for 48 h and immunoblotted for the indicated proteins.

b) After RNAi, cells were monitored by Incucyte time-lapse phase-contrast videomicroscopy to track cell confluency with respect to time (hours post-transfection, means, n = 6 wells, ± S.E.M.).

a

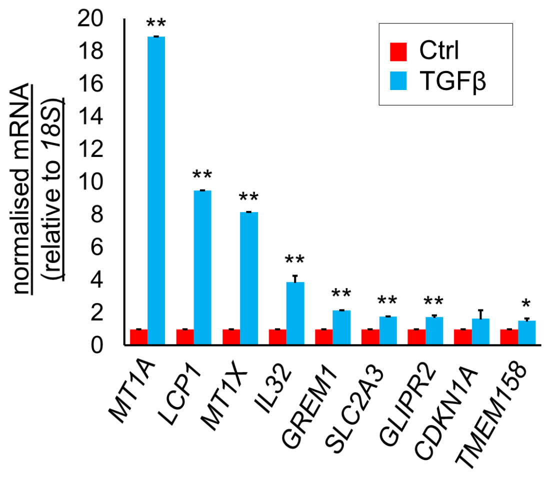

b

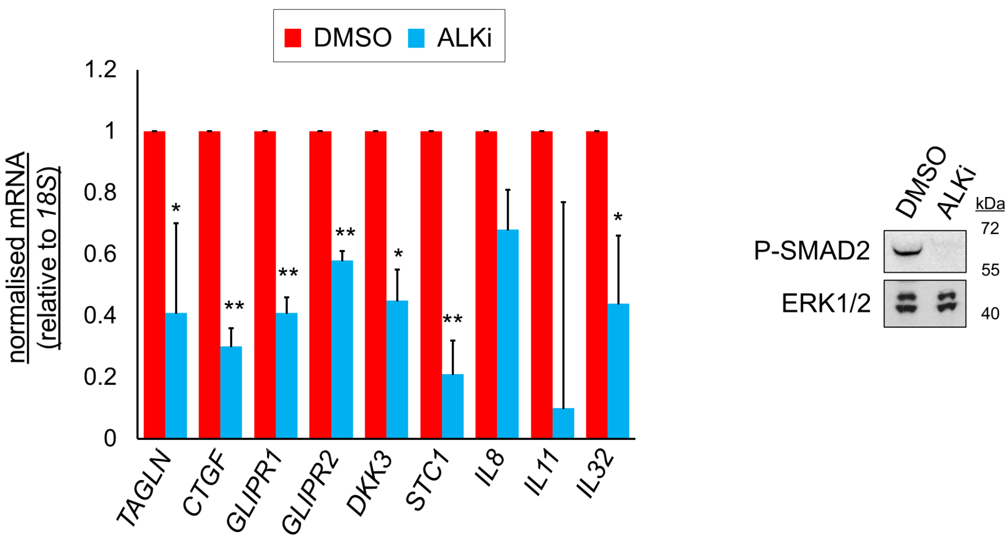

Supplementary Figure 2

a) In order to confirm the identity of a subset of less well-studied transcripts from the gene expression profiling as TGFβ-upregulated RNAs, A549 cells were either left untreated (Ctrl) or treated with 5 ng/ml TGFβ1 (TGFβ) for 16 h, and qRT-PCR was performed for the indicated transcripts (means, n = 3, ± S.D, * = $p < 0.05$ or ** = $p < 0.01$, two-tailed t-test).

b) A549 cells were treated with DMSO vehicle or 4 µM ALK inhibitor (ALKi) for 16 h, and qRT-PCR was then performed (means, n = 3, ± S.D, * = $p < 0.05$ or ** = $p < 0.01$, two-tailed t-test), or cells were lysed and immunoblotted for the indicated proteins (right panel, P-SMAD2 = phospho-serine 465/467 SMAD2, downstream readout of TGFβ-binding receptor serine-threonine kinase activity).

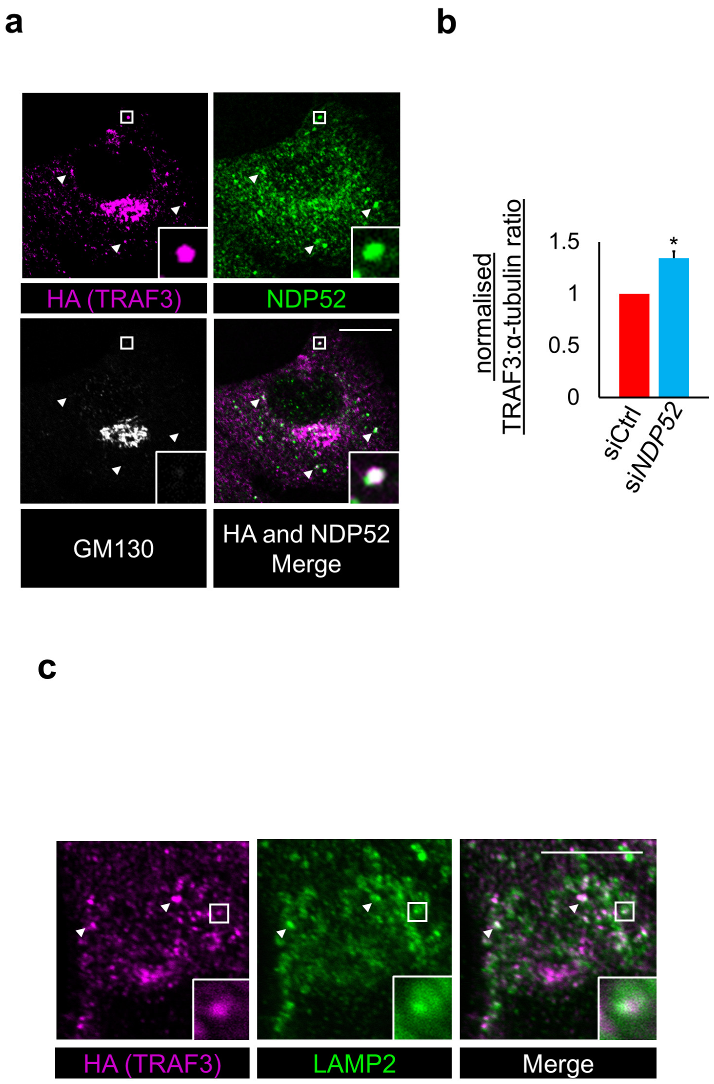

Supplementary Figure 3

a) A549 cells expressing FLAG-HA-TRAF3 were stained for the indicated epitopes and analysed by confocal microscopy (scale = 10 µm). Example TRAF3 foci are indicated with arrowheads. Boxes demarcate regions that are shown in zoomed insets.

b) A549 cells were transfected for 72 h with non-targeting control (siCtrl) or siRNA (si) targeting *NDP52*. Cell lysates were immunoblotted and blots quantified (mean of ratios to tubulin, normalised to siCtrl, n = 4, ± S. E. M., * $p < 0.05$, two-tailed t-test). A representative blot is shown in Figure 3f.

c) A549 cells stably expressing FLAG-HA-TRAF3 were treated with 100 nM BafA1 for 8 h, stained for the indicated epitopes, and analysed by confocal microscopy (scale = 5 µm). Dual-stained cytosolic foci are indicated with arrowheads and boxes demarcate areas shown in the zoomed inset.

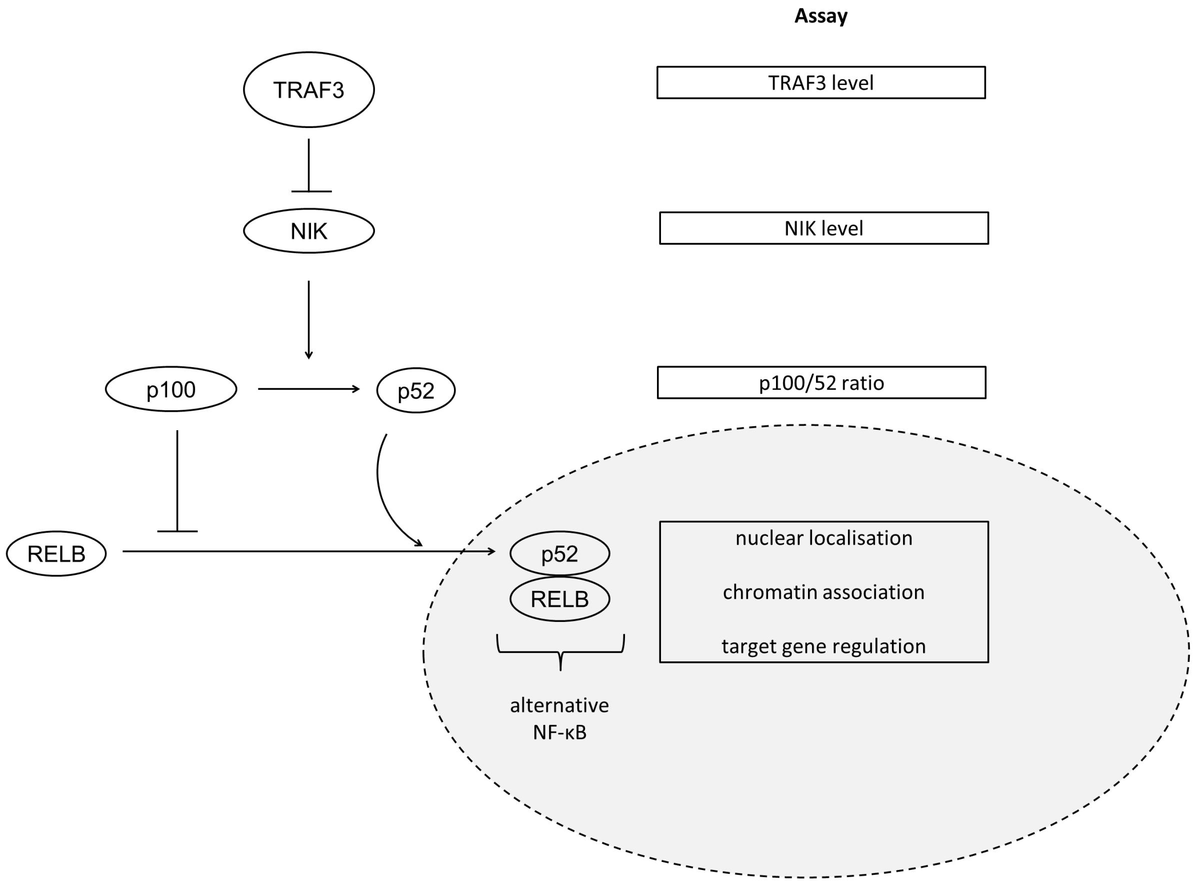

Supplementary Figure 4

Schematic diagram of the alternative NF-κB signaling cascade, along with the types of assays performed in this study. TRAF3 is the apical molecule in this pathway. It scaffolds degradative complexes that mediate proteolytic turnover of NF-κB-inducing kinase (NIK). TRAF3 activity results in reduced NIK levels in cell lysates.

NIK activity ordinarily results in processing of the NFκB2 protein (p100 precursor isoform) which typically acts to bind RELB and retain it in the cytosol of cells. The p52 product from NFκB2 processing retains binding to RELB but can now assist RELB to enter the nucleus and promote transcription. TRAF3 activity results in decreased processing of p100 to p52 and a change in ratio of these isoforms in cell lysates, as assayed by immunoblotting.

When RELB enters the nucleus it binds DNA. Conventionally, this occurs at NF-κB binding consensus elements in the promoters of direct target genes. RELB activity and binding to classic target genes or, hypothetically, other modes of association of RELB with chromatin, can be assayed by RELB chromatin immunoprecipitation. RELB can act as both a trans-activator and -repressor of gene expression.

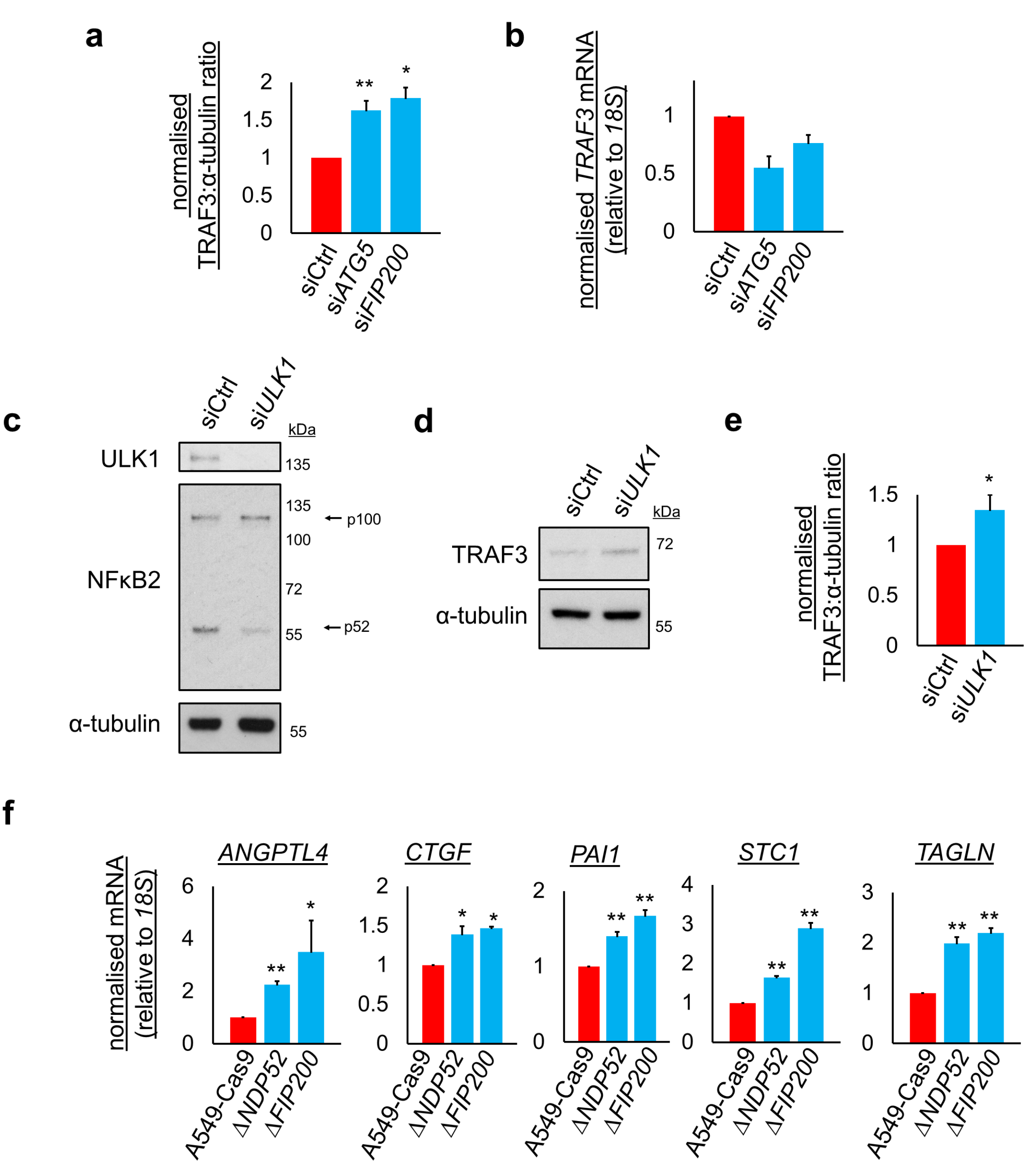

Supplementary Figure 5

a,b) A549 cells were transfected for 72 h with siCtrl or siRNA (si) targeting the indicated transcripts. a) Cell extracts were immunoblotted and blots quantified (mean of ratios to tubulin, normalised to siCtrl, n = 4, ± S. E. M., * p < 0.05, ** p < 0.01, two-tailed t-tests versus siCtrl). b) qRT-PCR was performed for the level of *TRAF3* mRNA (means, n = 3 technical replicates from one representative experiment, ± S.D.). A representative blot is shown in Figure 4a.

c-e) A549 cells were transfected with siCtrl or si*ULK1* for 72 h and immunoblotted for the indicated proteins. TRAF3 levels in d) are quantified in panel e) (mean ratios to tubulin, normalised to siCtrl, n = 4, ± S.E.M., * = p < 0.05, two-tailed t-test).

f) qRT-PCR was performed on A549-Cas9 control cells, A549 Δ*NDP52* and A549 Δ*FIP200* cells (means, n = 3, ± S. D., * p < 0.05, ** p < 0.01, two-tailed t-tests versus A549-Cas9).

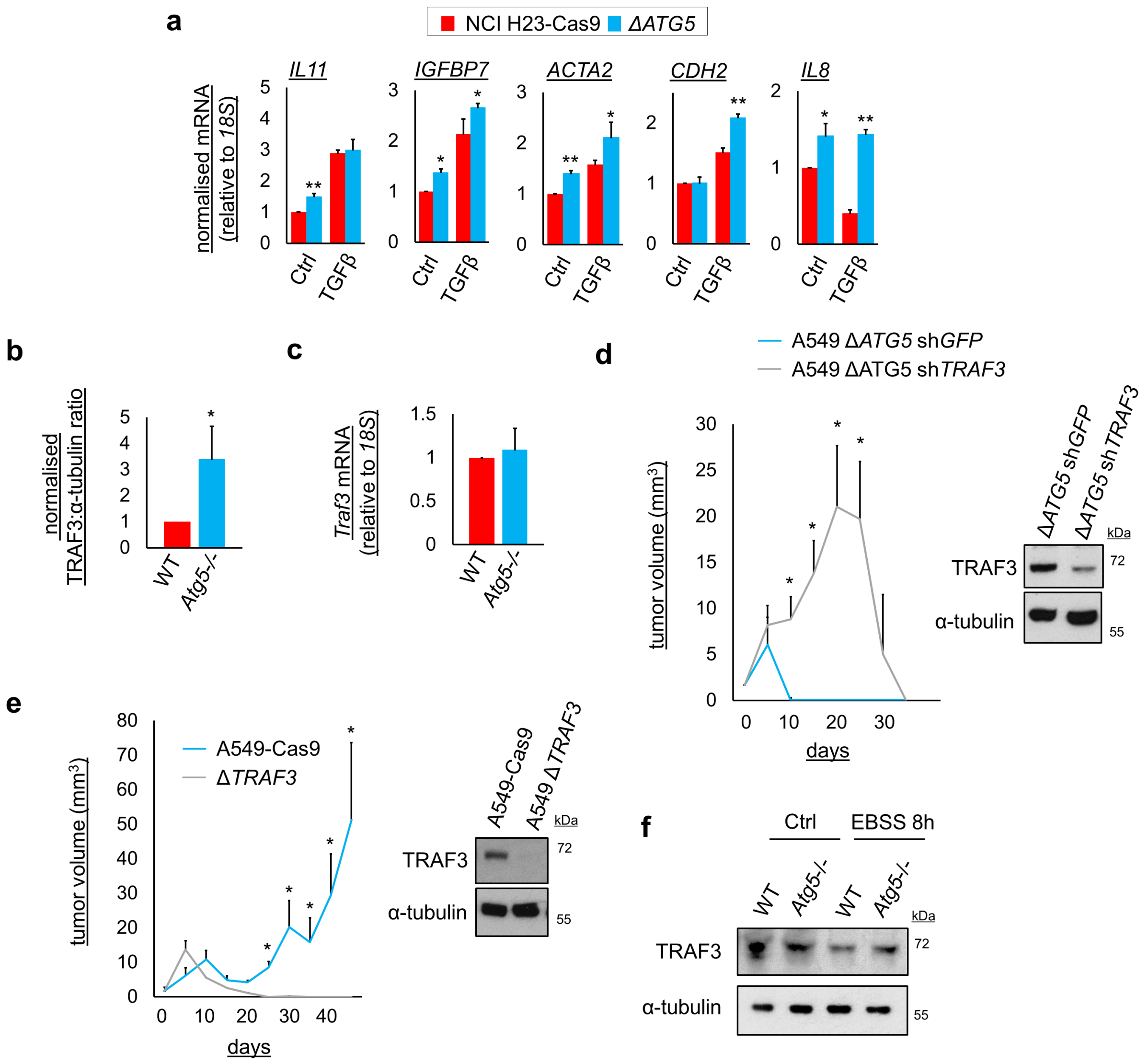

Supplementary Figure 6

a) qRT-PCR was performed on NCI-H23-Cas9 controls or NCI-H23 Δ*ATG5* cells (means, n = 3, ± S. D., * p < 0.05, ** p < 0.01, two-tailed t-tests versus NCI-H23-Cas9). **b,c)** Wild-type (WT) or *Atg5* null (*Atg5*-/-) KRAS V12 MEFs extracts were b) immunoblotted and blots quantified (mean ratios to tubulin, normalised to WT, n = 4, ± S. E. M., * p < 0.05, two-tailed t-test) or c) qRT-PCR was performed for the levels of *Traf3* mRNA (means, n = 3 technical replicates from one representative experiment, ± S.D.). A representative blot is shown in Figure 4h. **d)** A549 Δ*ATG5* cells were transduced with control non-targeting shRNA (sh*GFP*) or shRNA targeting *TRAF3* (sh*TRAF3*). Cells were subcutaneously injected into immunocompromised mice and tumor volume was monitored longitudinally (means, n = 6 flanks, ± S.E.M., * = p < 0.05 vs. shCtrl, two-tailed t-test). Stable lines were immunoblotted as indicated before injections. **e)** A549-Cas9 and cognate A549 Δ*TRAF3* cell lines were subcutaneously injected into immunocompromised mice, whereupon tumor volume was monitored longitudinally (means, n = 8 flanks, ± S.E.M., * = p < 0.05 vs. A549-Cas9, two-tailed t-test). Stable lines were immunoblotted as indicated before injections. Note that this experiment was performed as part of a larger set of xenograft experiments described in main Fig. 4e. **f)** WT or *Atg5*-/- MEFs (no KRAS) were amino acid and serum starved (EBSS 8h) and immunoblotted for TRAF3.

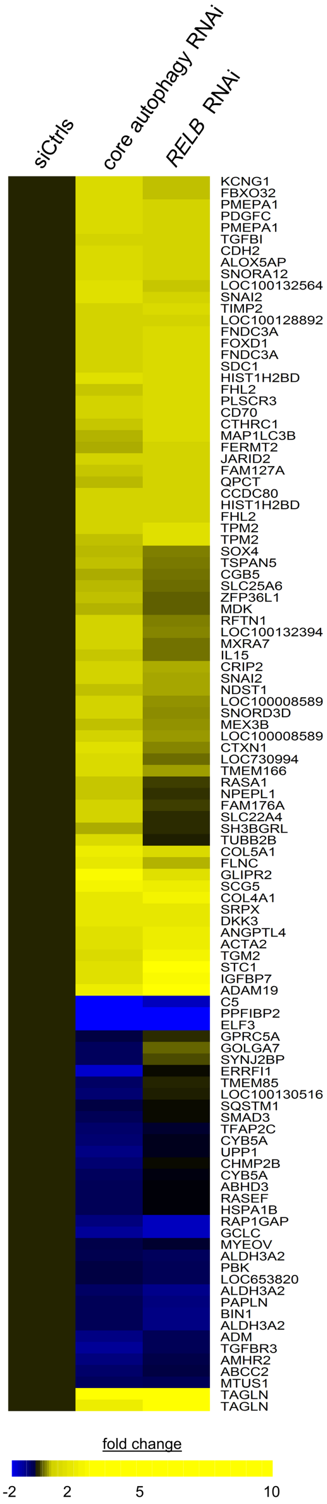

Supplementary Figure 7 Fold change heat map for individual gene probes that change upon autophagy inhibition, alongside the cognate fold change values for RELB inhibition. This enables comparison of transcript behaviour under both conditions. Autophagy-regulated genes were selected at a false-discovery rate of 0.1 and gated for a minimum fold change of 1.45 in either direction (Methods and Supplementary Dataset 1). Fold changes here are calculated by the quotient of the mean of all 6 replicates of autophagy-targeting siRNAs (3 biological replicates each of si*ATG5* and si*ULK1*) or, similarly, all 6 replicates of *RELB* targeting siRNAs (2 sequence-unrelated si*RELB* oligonucleotides), to the mean of the 6 non-targeting control replicates (siCtrls, 3 biological replicates of each of 2 sequence-unrelated control siRNAs).

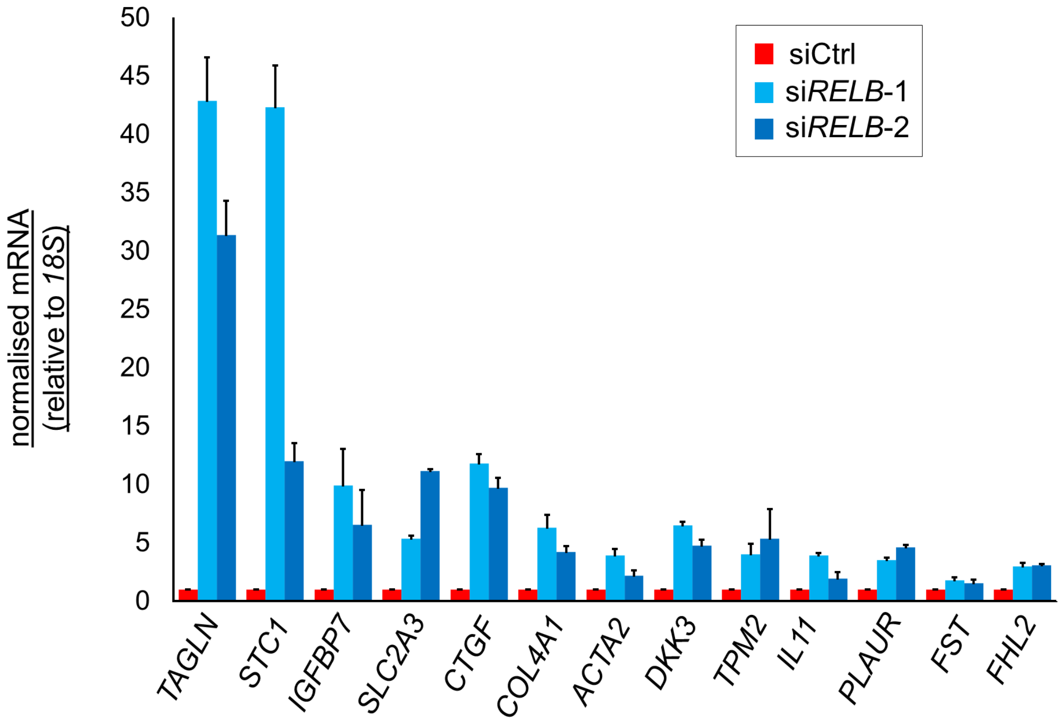

Supplementary Figure 8

A549 cells were transfected for 72 h with non-targeting control siRNA (siCtrl) or indicated siRNAs (si). qRT-PCR was performed for the indicated transcripts (means, n = 3 technical replicates, ± S.D.).

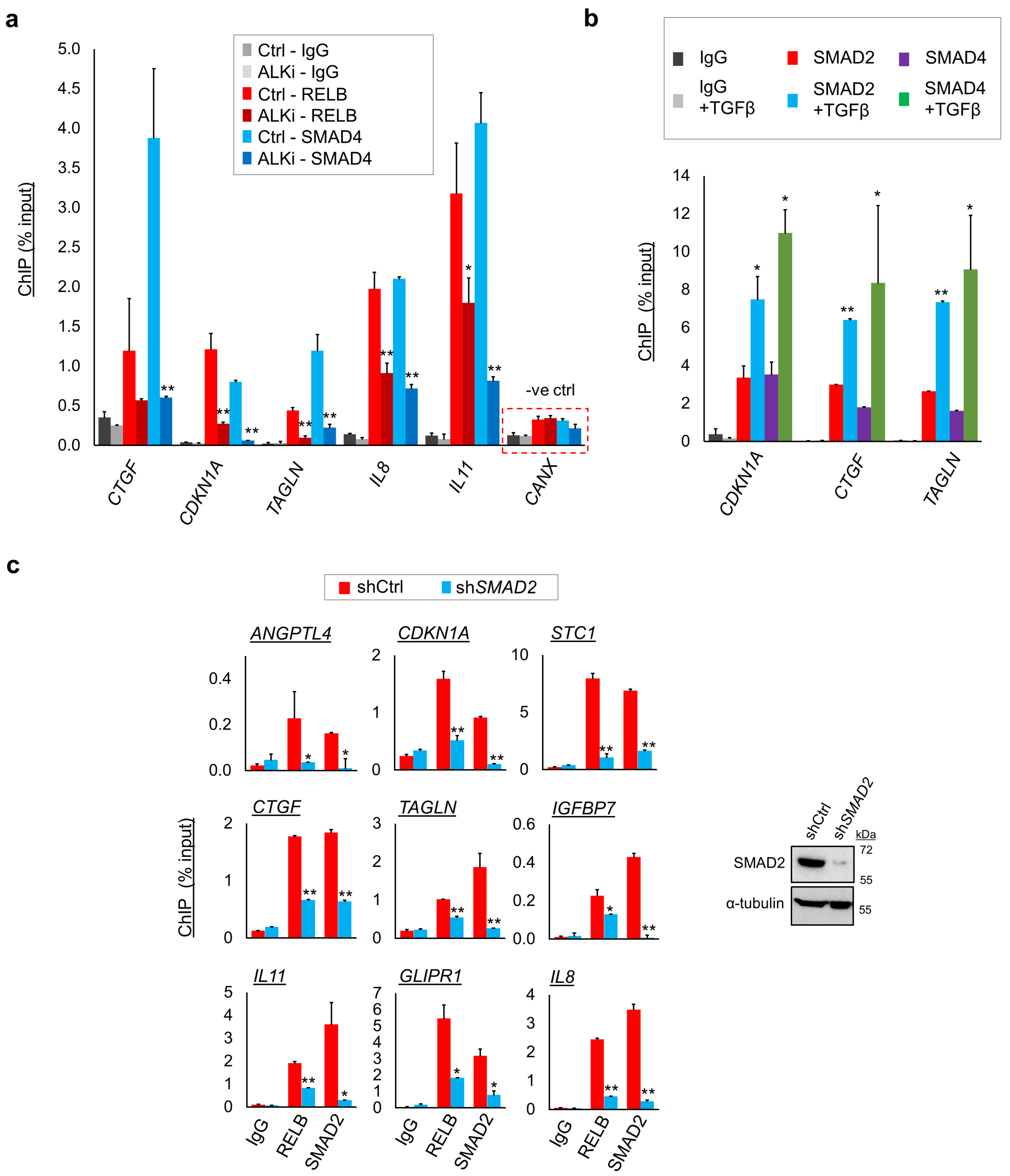

Supplementary Figure 9 **a)** A549 cells were treated with 4 µM ALK inhibitor (ALKi) or DMSO (Ctrl) for 16 h and then ChIP performed with the indicated antibody (shown above the chart). Gene names are shown below the chart (means, n = 3, ± S.D., * = p < 0.05 or ** = p < 0.01, vs. cognate Ctrl ChIP, two-tailed t-test). **b)** A549 cells were treated with 5 nM TGFβ for 16 h and then ChIP performed with the indicated antibody (shown above the chart). Gene names are shown below the chart (mean, n = 3, * = p < 0.05 or ** = p < 0.01, vs. cognate control [no TGFβ] condition, two-tailed t-test). **c)** A549 cells were transduced with shCtrl or sh*SMAD2* and stable pools selected. Pools were immunoblotted to confirm SMAD2 knockdown and ChIP performed with the indicated antibodies (shown below the charts). Gene names are above the charts (means, n = 3, ± S.D., * = p < 0.05 or ** = p < 0.01, vs. cognate shCtrl ChIP, two-tailed t-test).

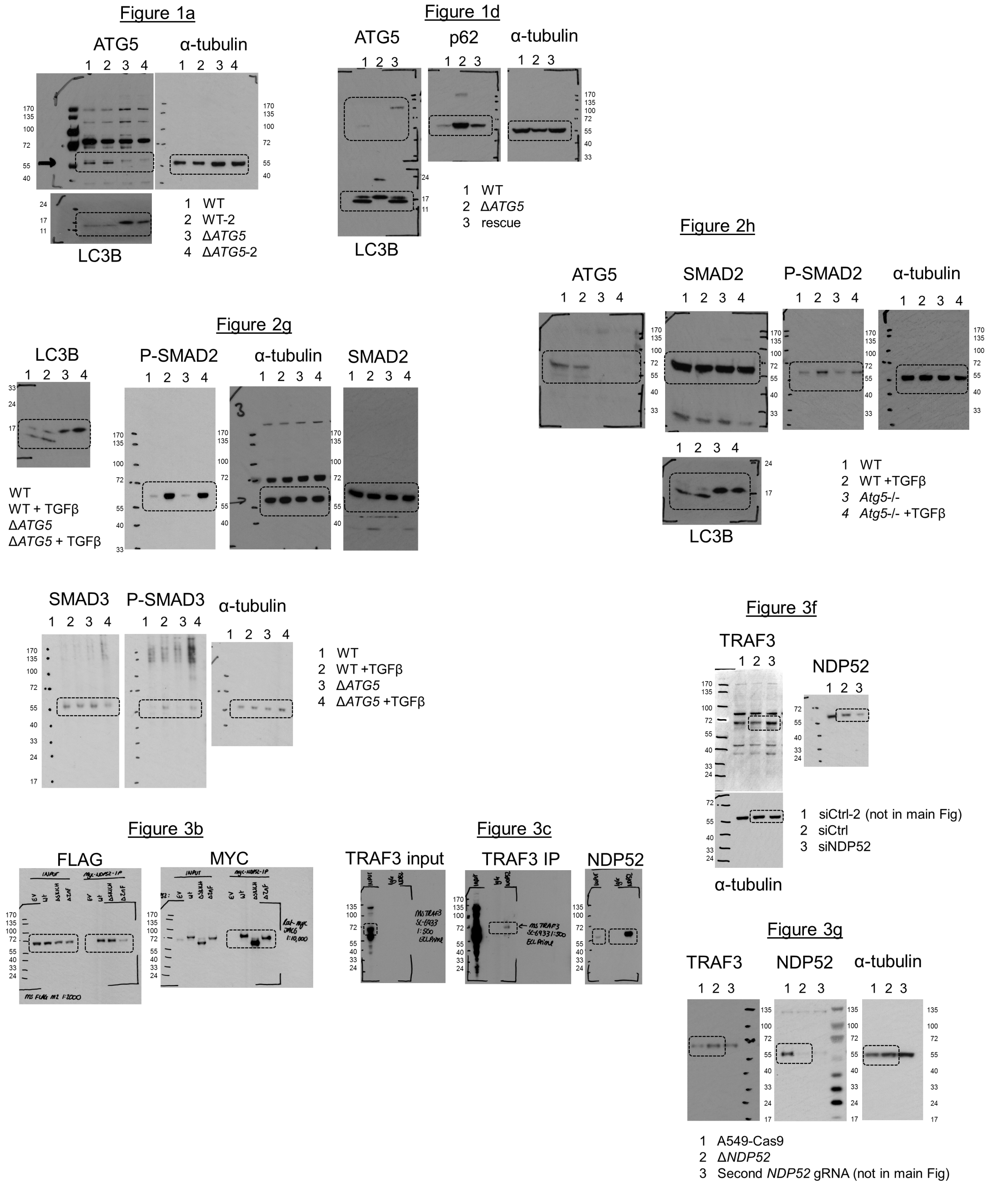

Supplementary Figure 10

Uncropped versions of blots presented in main Figures.

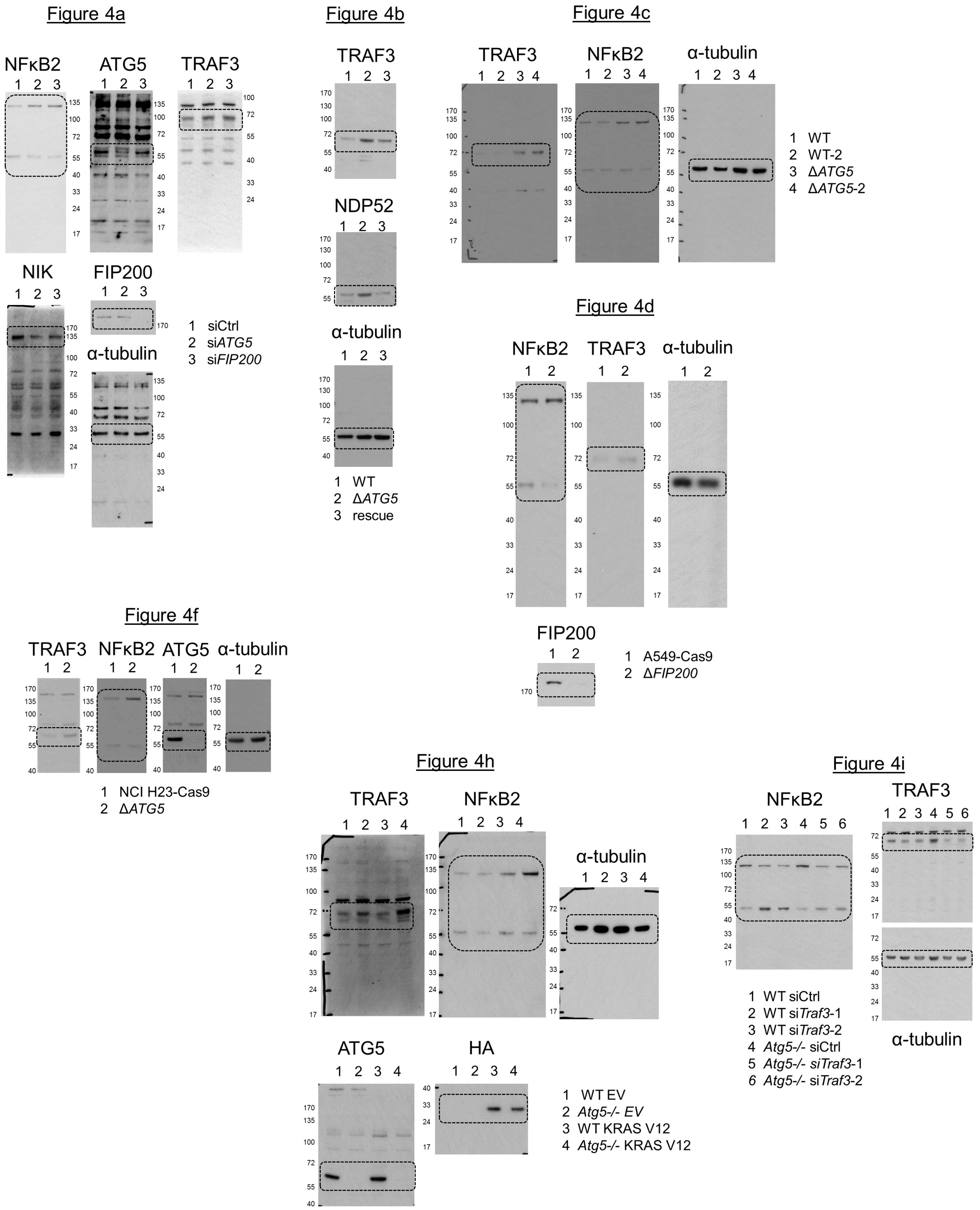

Supplementary Figure 10 (continued)

Figure 5a

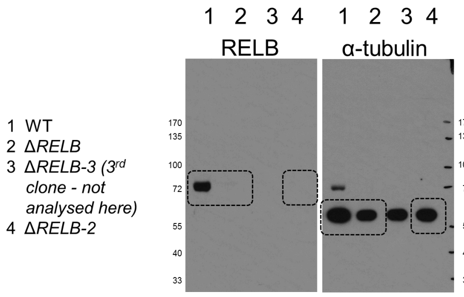

Figure 5g

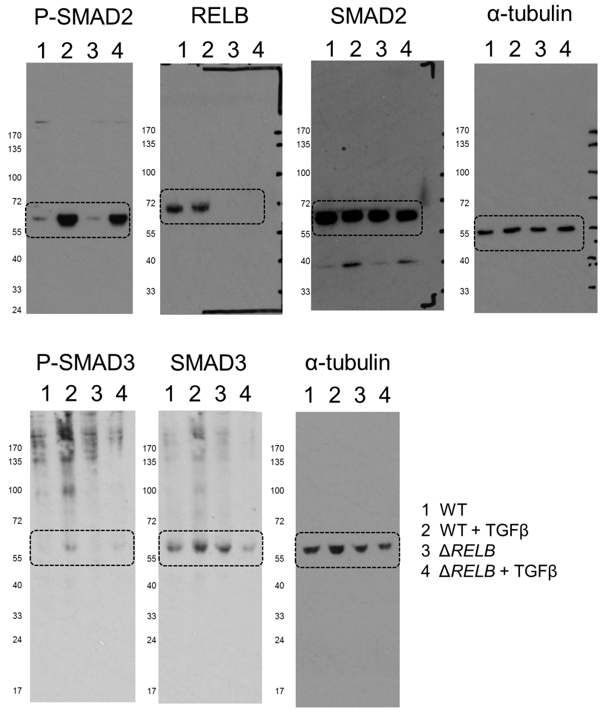

Figure 5c

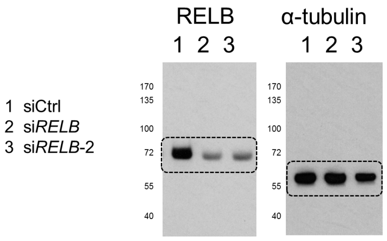

Figure 6b

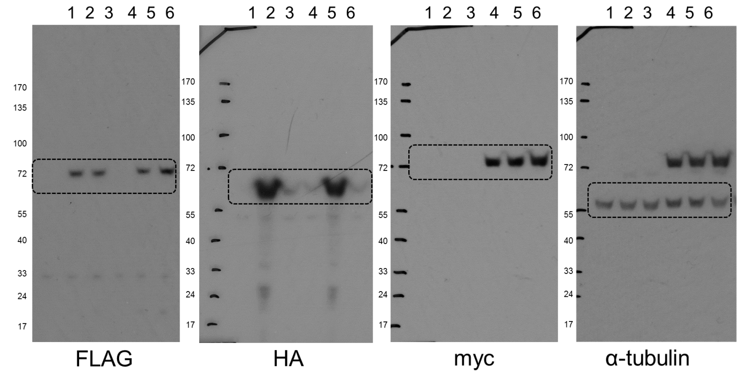

1-6) lanes 1-6 as shown left to right in main figure

Figure 6c

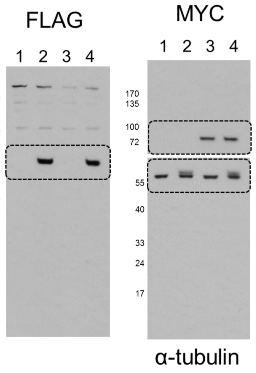

1-4) lanes 1-4 as shown left to right in main figure

Figure 6d

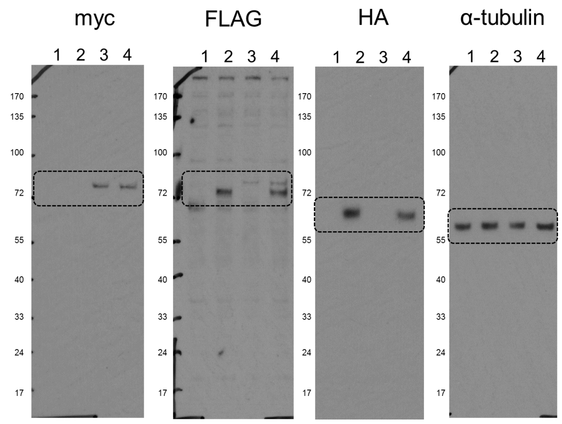

1-4) lanes 1-4 as shown left to right in main figure

Figure 6e

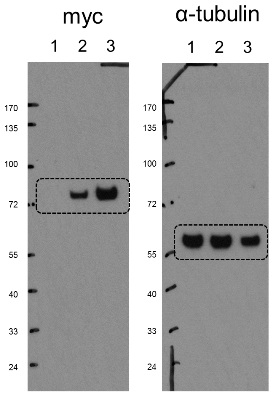

1-3) lanes 1-3 as shown left to right in main figure

Supplementary Figure 10 (continued)

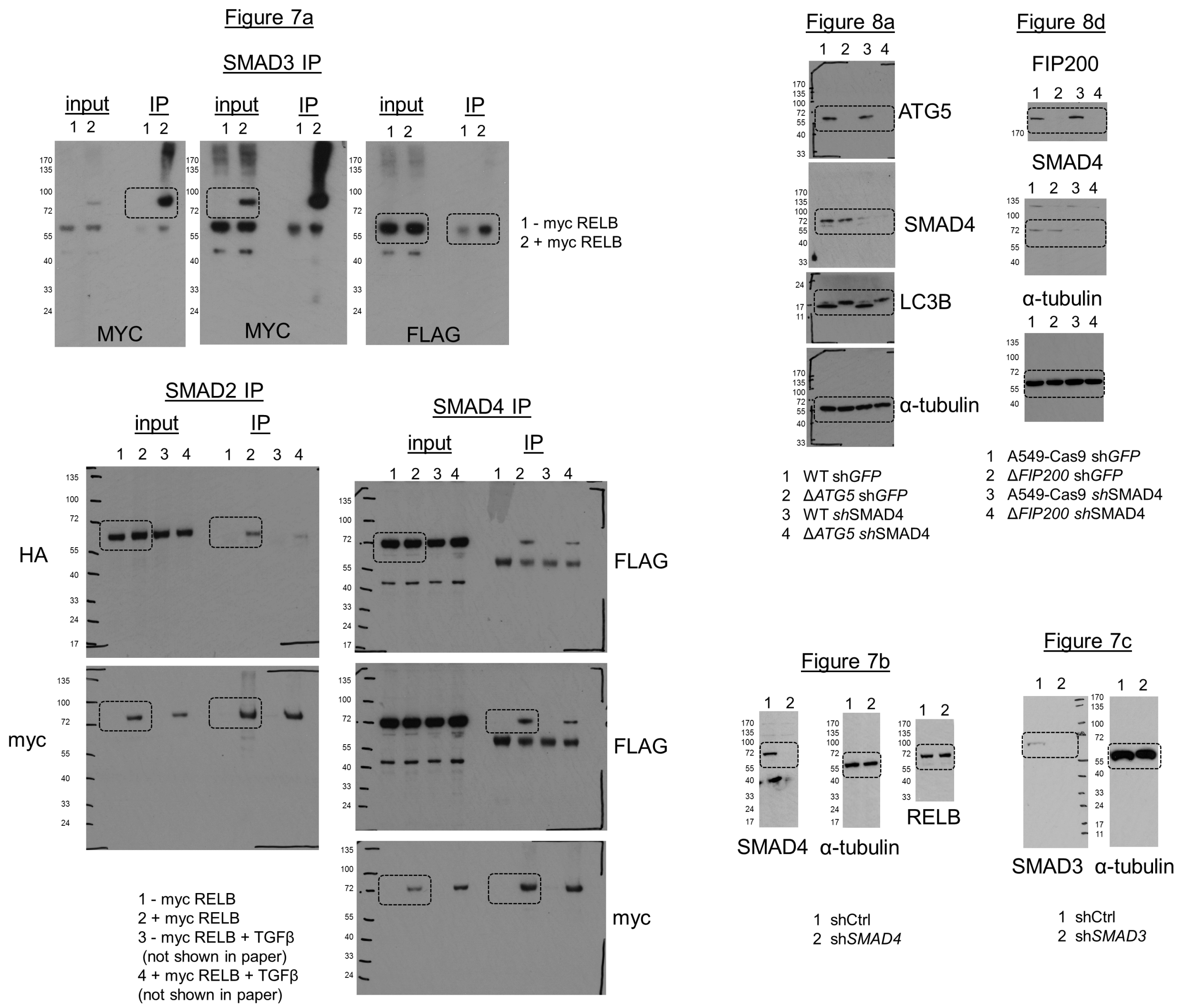

Supplementary Figure 10 (continued)

Supplementary Table 1

The top 50 most upregulated transcripts after *ATG5* and *ULK1* RNAi, based upon fold change criteria (also see Fig. 2b). Genes that are transactivated downstream of TGFβ signaling are annotated as **known** if this is evident from the existing literature, which is referenced here (Supplementary References). Genes are alternatively annotated with **confirmed here** if already known to be activated by TGFβ and where this is additionally verified within this study (e.g. Fig. 2c). If activation status is shown for the first time in this study (e.g. Supplementary Fig. S2a), the annotation reads **shown here**.

Of these TGFβ target genes, genes that are likely to be activated *directly*, as evidenced by SMAD binding to promoter regions, are highlighted in green. Here, **known** indicates identification of SMAD-binding by previous demonstration in the literature (Supplementary References). Alternatively, **confirmed here** refers to such genes where SMAD-binding is additionally verified by SMAD4 ChIP within this study (e.g. Fig. 7b, e, Supplementary Fig. 9a, b). Alternatively, **shown here** indicates that ChIP of SMAD4 to the promoters of these genes is shown for the first time in this study (e.g. Fig.7b, e).

N.B. the absence of annotation indicates that we present no evidence that a gene is regulated by TGFβ, but does not preclude the possibility.

Rank	Gene	Average fold repression by ATG5/ULK1	Transcript is TGFβ-driven?	Promoter binds SMAD2/3/4?	If known: reference(s)
1	*IL11*	3.2	known & confirmed here	known & confirmed here	1, 2
2	*NPTX1*	2.9	-	-	-
3	*TAGLN*	2.8	known & confirmed here	known & confirmed here	3
4	*GLIPR2*	2.7	shown here	-	-
5	*SCG5*	2.5	-	-	-
6	*ADAM19*	2.4	known	known	4
7	*COL5A1*	2.4	known	-	5
8	*FLNC*	2.3	-	-	-
9	*CGB1*	2.3	-	-	-
10	*SRPX*	2.3	-	-	-
11	*COL4A1*	2.2	known	-	6
12	*PAI1*	2.1	known & confirmed here	known	7
13	*DKK3*	2.1	-	-	-
14	*ANGPTL4*	2.1	known	known & confirmed here	8
15	*SNAI2*	2.0	known	known	9
16	*SPANXB1*	2.0	-	-	-
17	*FBXO32*	2.0	known	known	10
18	*FRMD6*	2.0	-	-	-
19	*CGB5*	2.0	-	-	-

20	*IGFBP7*	2.0	known & confirmed here	shown here	11
21	*CTXN1*	2.0	-	-	-
22	*SCARNA8*	2.0	-	-	-
23	*GLIPR1*	1.9	shown here	shown here	-
24	*ACTA2*	1.9	known	known	12
25	*PAPPA*	1.9	-	-	-
26	*GAL*	1.9	-	-	-
27	*HIST1H2BD*	1.9	-	-	-
28	*STC1*	1.9	shown here	-	-
29	*LOC100132564*	1.9	-	-	-
30	*SPANXB2*	1.9	-	-	-
31	*HIST1H2BK*	1.9	-	-	-
32	*SNORA12*	1.9	-	-	-
33	*EPHB1*	1.9	-	-	-
34	*SCARNA13*	1.8	-	-	-
35	*IL8*	1.8	known & confirmed here	shown here	1
36	*NLRP1*	1.8	-	-	-
37	*TMEM166*	1.8	-	-	-
38	*CDH2*	1.8	known	known	13
39	*ALOX5AP*	1.8	-	-	-
40	*PDGFC*	1.8	known	-	14
41	*EEF1A2*	1.8	-	-	-
42	*FXYD5*	1.8	-	-	-
43	*SLN*	1.8	-	-	-
44	*TGM2*	1.8	known	known	7, 15
45	*PMEPA1*	1.8	known	known	16, 17
46	*ARHGDIB*	1.8	-	-	-
47	*KCNG1*	1.8	-	-	-
48	*TP53INP1*	1.8	-	-	-
49	*CES1*	1.8	-	-	-
50	*PFKFB4*	1.8	-	-	-

Supplementary Table 2

The top 50 most upregulated transcripts after *RELB* RNAi, based upon fold change criteria (also see Fig. 5d). Genes that are transactivated downstream of TGFβ signaling are annotated as **known** if this is evident from the existing literature, which is referenced here (Supplementary References). Genes are alternatively annotated with **confirmed here** if already known to be activated by TGFβ and where this is additionally verified within this study (e.g. Fig. 5e). If activation status is shown for the first time in this study (e.g. Supplementary Fig. 2a), the annotation reads **shown here**.

Of these TGFβ target genes, genes that are likely to be activated *directly*, as evidenced by SMAD binding to promoter regions, are highlighted in green. Here, **known** indicates identification of SMAD-binding by previous demonstration in the literature (Supplementary References). Alternatively, **confirmed here** refers to such genes where SMAD-binding is additionally verified by SMAD4 ChIP within this study (e.g. Fig. 7b, e, Supplementary Fig. 9a, b). Alternatively, **shown here** indicates that ChIP of SMAD4 to the promoters of these genes is shown for the first time in this study (e.g. Fig.7b, e).

N.B. the absence of annotation indicates that we present no evidence that a gene is regulated by TGFβ, but does not preclude the possibility.

Rank	Gene	Average fold repression by RELB	Transcript is TGFβ-driven?	Promoter binds SMAD2/3/4?	If known: reference(s)
1	*TAGLN*	8.0	known & confirmed here	known & confirmed here	3
2	*GLIPR1*	4.4	shown here	shown here	-
3	*ACTG2*	3.5	known	-	18
4	*STC1*	3.4	shown here	-	-
5	*IL8*	3.2	known & confirmed here	shown here	1
6	*ADAM19*	3.1	known	known	4
7	*IGFBP7*	2.9	known & confirmed here	shown here	11
8	*ITGA5*	2.9	known	known	15, 19
9	*SLC2A3*	2.9	shown here	-	-
10	*LCP1*	2.8	shown here	-	-
11	*TMEM158*	2.6	shown here	-	-
12	*TGM2*	2.6	known	-	15
13	*COL4A1*	2.6	known	-	6
14	*CTGF*	2.6	known & confirmed here	known & confirmed here	20
15	*MT2A*	2.5	-	-	-
16	*MT1A*	2.5	shown here	-	-
17	*ANGPTL4*	2.4	known	known & confirmed here	8
18	*C17ORF91*	2.4	-	-	-

19	*SCG5*	2.4	-	-	-
20	*ACTA2*	2.3	known	known	12
21	*MT1X*	2.3	shown here	-	-
22	*SLC16A3*	2.2	-	-	-
23	*DKK3*	2.2	-	-	-
24	*SRPX*	2.1	-	-	-
25	*ZMAT3*	2.1	-	-	-
26	*IL32*	2.1	shown here	-	-
27	*SPOCD1*	2.1	-	-	-
28	*VCAN*	2.1	known	-	21
29	*TPM2*	2.1	known	-	22
30	*IL11*	2.1	known & confirmed here	known & confirmed here	1, 2
31	*PLAUR*	2.0	-	-	-
32	*CDKN1A*	2.0	known & confirmed here	known & confirmed here	23
33	*GLIPR2*	2.0	shown here	-	-
34	*COL22A1*	2.0	known	-	24
35	*MSN*	2.0	known	-	25
36	*CSRP1*	2.0	known	-	26
37	*HIST2H2AA3*	2.0	-	-	-
38	*FST*	2.0	known	-	27
39	*MAP1LC3A*	2.0	-	-	-
40	*TSPAN13*	1.9	-	-	-
41	*GREM1*	1.9	shown here	-	-
42	*PAI1*	1.9	known & confirmed here	known	7
43	*ZYX*	1.9	known	known	28
44	*LPXN*	1.9	-	-	-
45	*HERPUD1*	1.9	-	-	-
46	*KCNK6*	1.9	-	-	-
47	*DPYSL4*	1.9	-	-	-
48	*SH3KBP1*	1.9	-	-	-
49	*HIST1H2BD*	1.9	-	-	-
50	*ARF4*	1.9	-	-	-

Supplementary Table 3

Antibodies used in this study. Abbreviations used here: immunoblotting (IB), immunofluorescence (IF), immunoprecipitation (IP), immunohistochemistry (IHC) or chromatin immunoprecipitation (ChIP).

Antibody	Species	Application	Origin	Validation if not by manufacturer	Concentration if atypical
NIK	Rabbit poly	IB	CST #4994		1:1000
NFκB2	Rabbit poly	IB (human)	CST #3017		
NFκB2	Rabbit poly	IB (mouse)	CST #4882		1:1000
RELB	Rabbit mono	IB, IF	CST #4922	IF – reference 29	
RELB	Rabbit mono	ChIP	Millipore clone EP613Y #04-1077	RELB knockdown inhibits ChIP (not shown)	
LAMP2	Mouse mono	IF	Abcam #25631		
HA	Rat mono	IB, IF	Roche 3F10 11-8674-27001	IF – reference 29	1:400 for IF (0.1 μg/ml)
NDP52	Rabbit poly	IB, IP, IF	Abcam #68588	IP internally controlled Fig. 3c	1:500 for IF
NDP52	Mouse mono	IB, IF	Origene #501971		
H3me2K4	Rabbit poly	ChIP	Abcam #7766		
LC3B	Mouse mono	IF	Nanotools 2G6 #0260-100		
LC3B	Rabbit mono	IB	CST D11 #3868		
LC3B	Mouse mono	IHC	Nanotools 5F10 #0231-100	reference 30	1:50
α-tubulin	Mouse mono	IB	Sigma #T9026		1:50 000
myc	Rabbit mono	IB	CST 71D10 #2278		
myc	Mouse mono	IB	Upstate 4A6 #05724		
FLAG	Rat mono	IB	Agilent #200473		
FLAG (M2)	Mouse mono	IB	Agilent #200471		
Paxillin	Mouse mono	IF	BD 165 #610619		1:400
TRAF3	Rabbit poly	IB	Sigma #HPA002933		1:1000
GM130	Mouse mono	IF	BD 35/GM130 #610822		0.25 μg/ml
ATG5	Rabbit poly	IB	Sigma #A0731		1:1000
ATG5	Rabbit poly	IB	CST #2630		
ATG5	Mouse mono	IB	Sigma 3D2 #A0856		
FIP200	Rabbit mono	IB	CST D10D11 #12436		
p62	Rabbit poly	IB	Enzo #BML-PW9860		1:5000
ERK1/2	Rabbit poly	IB	CST #9102		
SMAD2	Rabbit mono	ChIP, IB	CST D43B4 #5339		
SMAD4	Rabbit poly	ChIP, IB	CST #9515		
P-SMAD2 (465/467)	Rabbit mono	IB	CST 138D4 #3108		1:1000
P-SMAD3 (423/5)	Rabbit mono	IB	CST C25A9 #9520		1:1000
SMAD3	Rabbit mono	IB	CST C67H9 #9523		1:500
ULK1	Rabbit mono	IB	CST D8H5 #8054		
IgG –ve ctrl	Rabbit poly	IP, ChIP	CST #2729		

Supplementary Table 4 Details of plasmids used in this study.

Plasmid	Source
MSCV NTAP-*TRAF3*-IRES-PURO	Gateway cloning from pDONR223 into MSCV DEST vector.
MSCV NTAP-*KRAS*^{G12V}-IRES PURO	Gateway cloning from pDONR223 into MSCV DEST vector.
MSCV NTAP-EV	Gateway cloning from pDONR223 into MSCV DEST vector.
MSCV NTAP-DEST IRES PURO	As used previously (ref 31).
pdcDNA FLAG DEST	An N-ter FLAG-tagged DEST version of pcDNA (F. Van Roy and B. Janssens).
pdcDNA myc DEST	An N-ter 6x(myc)-tagged DEST version of pcDNA (F. Van Roy and B. Janssens).
pDONR223 EV	A short in-frame stop codon sequence cloned into pDONR223.
pDONR223 NDP52 (and Δ)	Human NDP52 was PCR amplified from cDNA.
pDONR223 *KRAS*^{G12V}	*KRAS*^{G12V} was amplified from pBabe puro *KRAS* V12 (M. Ditzel).
pDONR223 *TRAF3*	*TRAF3* was amplified from pcDNA-HA-TRAF3, a gift of Shao-Cong Sun (Addgene #44032) (ref 32).
pDONR223 *RELB*	RELB was PCR amplified from cDNA.
pdcDNA FLAG-*TRAF3*	Gateway cloning from pDONR223 into pdcDNA FLAG DEST.
pBabe puro *GFP-ATG5*	Gift from Kevin Ryan.
pdcDNA myc-*NDP52* (and Δ)	Gateway cloning from pDONR223 into pdcDNA myc DEST.
pdcDNA myc EV	Gateway cloning from pDONR223 into pdcDNA myc DEST.
pdcDNA myc *RELB*	Gateway cloning from pDONR223 into pdcDNA myc DEST.
pdcDNA myc *RELB* AA	Site-directed mutagenesis on pdcDNA myc *RELB* to *R141A* Y142A.
pCMV5 *SMAD2*-HA	Gift from Joan Massague (Addgene #14930) (ref 33)
pRK5F Smad3	Gift from Rik Derynck (Addgene #12625) (ref 34)
pcDNA FLAG-*SMAD4*	Gift from Joan Massague (Addgene #14959) (ref 35)
pGL3 $(CAGA)_{12}$-luciferase reporter	Gift from Peter ten Dijke, a firefly luciferase reporter with 12 repeats of the SMAD response element (AGCCAGACA) (ref 36)
pNF3 luciferase reporter	A firefly luciferase reporter plasmid containining three tandem repeats of an NF-κB binding consensus element. (ref 37)
pGL3-basic	A promoterless firefly luciferase reporter plasmid. Promega.
pRL-SV40	A *Renilla* luciferase control reporter vector. Promega.
pLKO.1 hygro	pLKO.1 hygro was a gift from Bob Weinberg (Addgene # 24150)
pLKO.1 puro shNTC	As described (ref 29).
pLKO.1 puro sh*GFP*	Target ACAACAGCCACAACGTCTATA
pLKO.1 puro sh*SMAD4*	Target CGAGTTGTATCACCTGGAATT, a gift of Peter Ten Dijke.

pLKO.1 puro sh*SMAD2*	A gift of Peter Ten Dijke.
pLKO.1 hygro sh*GFP*	Target ACAACAGCCACAACGTCTATA
pLKO.1 hygro sh*SMAD4*	Target CGAGTTGTATCACCTGGAATT
pRetroSuper puro shCtrl	Target TAAGGCTATGAAGAGATAC
pRetroSuper puro sh*SMAD3*	A gift from Joan Massague (Addgene plasmid # 15726) (ref 38)
pLKO.1 hygro sh*TRAF3*	Target CCTGCTTCCTTGGCCGTTTAA
gRNA	A gift from George Church (Addgene #41824) (ref 39)
pSpCas9(BB)-2A-Puro v2.0	A gift from Feng Zhang (Addgene #62988) (ref 40)
lentiCas9-BLAST	Gift from Feng Zhang (Addgene #52962) (ref 41)
lentiGuide-Puro	Gift from Feng Zhang (Addgene #52963) (ref 41)

Supplementary Table 5 DNA oligonucleotide sequences of primers used in this study.

Hs/Mm 18S	GTAACCCGTTGAACCCCATT	CCATCCAATCGGTAGTAGCG
Hs TRAF3	TCCTTGTTGCAGAATGAAAG	ATCACTCGCTGTAAATGAAG
Hs CTGF	GTTACCAATGACAACGCCTC	TTGCCCTTCTTAATGTTCTCTTCC
Hs CDH2	TCCCATCATAATCACAGATTCGG	AACATCAGCACAAGGATAAGCAG
Hs TAGLN	AAGAATGATGGGCACTACCG	ACTGATGATCTGCCGAGGTC
Hs CDKN1A	TGCCGAAGTCAGTTCCTTGT	GTTCTGACATGGCGCCTCC
Hs IL8	AGATCTGAAGTGTGATGACTCAGG	GAAGCTTGTGTGCTCTGCTGTCTC
Hs IL11	CATGAACTAGGGACAAATTCCCA	CAGGTAGGACAGTAGGTCCG
Hs IL32	AATGCAAAATGCAGAATCAG	GTAGAGGAGTGAGCTCTG
Hs IGFBP7	AAGGACATCTGGAATGTCACTG	CTTAGAGGAGATACCAGCACCC
Hs SLC2A3	TGCTTAGGAGAGACCGAGTGA	ATATCAGAACCCAAGGGAGGA
Hs COL4A1	TGTTGGCTATCCAGGAAGTC	CACCCTTTGAACCTTTGTCTC
Hs ACTA2	ACCATGAAGATCAAGATCATTGCC	CATTTGCGGTGGACAATGGA
Hs DKK3	GTGGAAGAGATGGAGGCAG	AGTCTGGTTGTTGGTTATCTTGTG
Hs TPM2	ACAAGAAGCAAGCTGAGGAC	CATCTGCCTCAGCATCAGTG
Hs PLAUR	CTTGTGGGAAGAAGGAGAAGAG	GTAACGGCTTCGGGAATAGG
Hs FST	AAGACCGAACTGAGCAAGGA	TTCTCACACGTTTCTTTACAGGG
Hs MT1A	GTGCGCCTTATAGCCTCTCA	AGGAGCAGCAGCTCTTCTTG
Hs ACTG2	CAGCAGCTTCCTCTTCCTCC	CAGCGGACTCCATGCCAATA
Hs ITGA5	TCTATGAGCTGAGAAACAATGG	AACCCAAAGTGTGAATATCTCC
Hs MT2A	GCACCTCCTGCAAGAAAAGCTG	CGGTCACGGTCAGGGTTGTA
Hs TMEM158	GGCTGAACCGTAAGCCCATT	CTCCACACCACGATGACCAG
Hs STC1	AAACTCAGCTGAAGTGGTTCG	ACATTCAGCTTGCTGTAGCAC
Hs MT1X	GCGTGTTTTCCTCTTGATCGG	AGGAGCCAACAGGCGAGC
Hs PAI1	CCGTTGAAGTAGAGGGCATT	CCACTTCTTCAGGCTGTTCC
Hs GLIPR1	TCTTCCGCCATCACAAACTG	CTGCCCAAACAACCTGAGTG
Hs LCP1	GTGGCCAGAAGGTCAATGAT	TTTTTCGGGCCATAGAGATG
Hs GLIPR2	GGCTCAACAGTATTCTGAGG	ACTGTACCATCTATCAGCCA
Hs ADAM19	GCATCGTTTCCCAGGACTTCTC	CCAGCCCTCTGTGATCTGTATTCT
Hs TGM2	TCCAACCTCATCAAGGTGCG	GTCTGGGATCTCCACCGTCT
Hs GAPDH	GGAGCCAAACGGGTCATCATCTC	GAGGGGCCATCCACAGTCTTCT
Hs ANGPTL4	GACGAGATGAATGTCCTGGC	CCTTGAGTTGTGTCTGCAGG
Hs ZYX	GCAGAATGTGGCTGTCAACGAAC	TGAAGCAGGCGATGTGGAACAG
Hs SLC27A2	TTTCAGCCAGCCAGTTTTG	TCTCCTCGTAAGCCATTTCC
Hs SLC3A1	CCCAAGGAGGTGCTGTTCC	TGAATACCTTTCAGATCTCCGTTCC
Hs GREM1	GCACTGACAGTATGAGCCGC	GAAGCGGTTGATGATGGTGC
Hs FHL2	CATGAGCAGGGAGGATAGGG	CTGTGAGCTGGGAAATGTGG
Mm Traf3	GACTCTTCTAAGGAGTGAGG	TGGATGCTCTTGTTTTTCTC
Mm Tagln	GCTACTCTCCTTCCAGTCCA	CAATTTGCTCAGAATCACACCA
Mm Ctgf	GTGCACTGCCAAAGATGGT	GGGCCAAATGTGTCTTCCA
Mm Igfbp7	CTGGGTGCTGGTATCTCCTC	TGGCTGTAATAAAGTGTTAGTGGG
Mm Pai2	ACCTGTCCAGATGATGTTCC	TTCACTTTCCAGCAATTCCA
Mm Angptl4	TCTTCAGAGCCAGATAGACCT	CAATGAGCTGGGTCATCTTGG
Mm Glipr1	TCACGGATACACCCAAATTTCAC	GTAATTTCCTGCTGGTCCATAGTC
**ChIP target**		
CDKN1A	GGAGGCAAAAGTCCTGTGTTC	GGAAGGAGGGAATTGGAGAG
TAGLN	AGTGGGGGAGGCTGACAT	TCGCAGGAAGGAGTGAAGAC
IL8	GATGAGGGTGCATAAGTTCTCTAG	TCTTCCTGGCTCTTGTCCTAG
IL11	AGCCTGAGTGTCTGCTCCG	TGACACATCCTGACTCACCCTCC
CANX	TGGGCCACTTCCATTTTGTG	CAGGGCAGAGAGATACAGGG
CTGF	ATATGAATCAGGAGTGGTGCGA	CAACTCACACCGGATTGATCC
GLIPR1	AGGAAGTCTGACACAGCCTC	AGGCAAATCAGAAGAAGCGG
ANGPTL4	CCTTACTGGATGGGAGGAAAG	CCCAGAGTGACCAGGAAGAC
IGFBP7	GATTGGAGGATGTTTCCC	CATGTCACATTGTGGTTCTT
STC1	GGAACCGGTACCTCGAATCT	GTCGCCTCCCTTCCTAGTTT

Supplementary References

1. Gupta J, Robbins J, Jilling T, Seth P. TGFbeta-dependent induction of interleukin-11 and interleukin-8 involves SMAD and p38 MAPK pathways in breast tumor models with varied bone metastases potential. *Cancer biology & therapy* **11**, 311-316 (2011).

2. Kang Y*, et al.* Breast cancer bone metastasis mediated by the Smad tumor suppressor pathway. *Proceedings of the National Academy of Sciences of the United States of America* **102**, 13909-13914 (2005).

3. Yu H*, et al.* Transgelin is a direct target of TGF-beta/Smad3-dependent epithelial cell migration in lung fibrosis. *FASEB journal : official publication of the Federation of American Societies for Experimental Biology* **22**, 1778-1789 (2008).

4. Chan MW*, et al.* Aberrant transforming growth factor beta1 signaling and SMAD4 nuclear translocation confer epigenetic repression of ADAM19 in ovarian cancer. *Neoplasia* **10**, 908-919 (2008).

5. Cheon DJ*, et al.* A collagen-remodeling gene signature regulated by TGF-beta signaling is associated with metastasis and poor survival in serous ovarian cancer. *Clinical cancer research : an official journal of the American Association for Cancer Research* **20**, 711-723 (2014).

6. Li J, Qu X, Ricardo SD, Bertram JF, Nikolic-Paterson DJ. Resveratrol inhibits renal fibrosis in the obstructed kidney: potential role in deacetylation of Smad3. *The American journal of pathology* **177**, 1065-1071 (2010).

7. Koinuma D, Tsutsumi S, Kamimura N, Imamura T, Aburatani H, Miyazono K. Promoter-wide analysis of Smad4 binding sites in human epithelial cells. *Cancer science* **100**, 2133-2142 (2009).

8. Ferguson BW*, et al.* The cancer gene WWOX behaves as an inhibitor of SMAD3 transcriptional activity via direct binding. *BMC cancer* **13**, 593 (2013).

9. Brandl M*, et al.* IKK(alpha) controls canonical TGF(ss)-SMAD signaling to regulate genes expressing SNAIL and SLUG during EMT in panc1 cells. *Journal of cell science* **123**, 4231-4239 (2010).

10. Qin H*, et al.* An integrative ChIP-chip and gene expression profiling to model SMAD regulatory modules. *BMC systems biology* **3**, 73 (2009).

11. Rao C, Lin SL, Ruan WJ, Wen H, Wu DJ, Deng H. High expression of IGFBP7 in fibroblasts induced by colorectal cancer cells is co-regulated by TGF-beta and Wnt signaling in a Smad2/3-Dvl2/3-dependent manner. *PloS one* **9**, e85340 (2014).

12. Hu B, Wu Z, Phan SH. Smad3 mediates transforming growth factor-beta-induced alpha-smooth muscle actin expression. *American journal of respiratory cell and molecular biology* **29**, 397-404 (2003).

13. Kang Y*, et al.* SMAD4 regulates cell motility through transcription of N-cadherin in human pancreatic ductal epithelium. *PloS one* **9**, e107948 (2014).

14. Wang Y, Serra R. PDGF mediates TGFbeta-induced migration during development of the spinous process. *Developmental biology* **365**, 110-117 (2012).

15. Ranganathan P*, et al.* Expression profiling of genes regulated by TGF-beta: differential regulation in normal and tumour cells. *BMC genomics* **8**, 98 (2007).

16. Brunschwig EB*, et al.* PMEPA1, a transforming growth factor-beta-induced marker of terminal colonocyte differentiation whose expression is maintained in primary and metastatic colon cancer. *Cancer research* **63**, 1568-1575 (2003).

17. Mullen AC*, et al.* Master transcription factors determine cell-type-specific responses to TGF-beta signaling. *Cell* **147**, 565-576 (2011).

18. Zavadil J*, et al.* Genetic programs of epithelial cell plasticity directed by transforming growth factor-beta. *Proceedings of the National Academy of Sciences of the United States of America* **98**, 6686-6691 (2001).

19. Isogaya K*, et al.* A Smad3 and TTF-1/NKX2-1 complex regulates Smad4-independent gene expression. *Cell research* **24**, 994-1008 (2014).

20. Grotendorst GR, Okochi H, Hayashi N. A novel transforming growth factor beta response element controls the expression of the connective tissue growth factor gene. *Cell growth & differentiation : the molecular biology journal of the American Association for Cancer Research* **7**, 469-480 (1996).

21. Yeung TL*, et al.* TGF-beta modulates ovarian cancer invasion by upregulating CAF-derived versican in the tumor microenvironment. *Cancer research* **73**, 5016-5028 (2013).

22. Bakin AV, Safina A, Rinehart C, Daroqui C, Darbary H, Helfman DM. A critical role of tropomyosins in TGF-beta regulation of the actin cytoskeleton and cell motility in epithelial cells. *Molecular biology of the cell* **15**, 4682-4694 (2004).

23. Araki S*, et al.* TGF-beta1-induced expression of human Mdm2 correlates with late-stage metastatic breast cancer. *The Journal of clinical investigation* **120**, 290-302 (2010).

24. Zhang J*, et al.* Sorafenib inhibits epithelial-mesenchymal transition through an epigenetic-based mechanism in human lung epithelial cells. *PloS one* **8**, e64954 (2013).

25. Valcourt U, Kowanetz M, Niimi H, Heldin CH, Moustakas A. TGF-beta and the Smad signaling pathway support transcriptomic reprogramming during epithelial-mesenchymal cell transition. *Molecular biology of the cell* **16**, 1987-2002 (2005).

26. Bianchi-Smiraglia A, Kunnev D, Limoge M, Lee A, Beckerle MC, Bakin AV. Integrin-beta5 and zyxin mediate formation of ventral stress fibers in response to transforming growth factor beta. *Cell cycle* **12**, 3377-3389 (2013).

27. Bartholin L*, et al.* Transcription activation of FLRG and follistatin by activin A, through Smad proteins, participates in a negative feedback loop to modulate activin A function. *Oncogene* **21**, 2227-2235 (2002).

28. Mise N, Savai R, Yu H, Schwarz J, Kaminski N, Eickelberg O. Zyxin is a transforming growth factor-beta (TGF-beta)/Smad3 target gene that regulates lung cancer cell motility via integrin alpha5beta1. *The Journal of biological chemistry* **287**, 31393-31405 (2012).

29. Newman AC*, et al.* TBK1 kinase addiction in lung cancer cells is mediated via autophagy of Tax1bp1/Ndp52 and non-canonical NF-kappaB signalling. *PloS one* **7**, e50672 (2012).

30. Rosenfeldt MT, Nixon C, Liu E, Mah LY, Ryan KM. Analysis of macroautophagy by immunohistochemistry. *Autophagy* **8**, 963-969 (2012).

31. Behrends C, Sowa ME, Gygi SP, Harper JW. Network organization of the human autophagy system. *Nature* **466**, 68-76 (2010).

32. Liao G, Zhang M, Harhaj EW, Sun SC. Regulation of the NF-kappaB-inducing kinase by tumor necrosis factor receptor-associated factor 3-induced degradation. *The Journal of biological chemistry* **279**, 26243-26250 (2004).

33. Hata A, Lo RS, Wotton D, Lagna G, Massague J. Mutations increasing autoinhibition inactivate tumour suppressors Smad2 and Smad4. *Nature* **388**, 82-87 (1997).

34. Zhang Y, Feng XH, Derynck R. Smad3 and Smad4 cooperate with c-Jun/c-Fos to mediate TGF-beta-induced transcription. *Nature* **394**, 909-913 (1998).

35. Kang Y*, et al.* Breast cancer bone metastasis mediated by the Smad tumor suppressor pathway. *Proceedings of the National Academy of Sciences of the United States of America* **102**, 13909-13914 (2005).

36. Dennler S, Itoh S, Vivien D, ten Dijke P, Huet S, Gauthier JM. Direct binding of Smad3 and Smad4 to critical TGF beta-inducible elements in the promoter of human plasminogen activator inhibitor-type 1 gene. *EMBO J* **17**, 3091-3100 (1998).

37. Roumestan C*, et al.* Anti-inflammatory properties of desipramine and fluoxetine. *Respir Res* **8**, 35 (2007).

38. He W, Dorn DC, Erdjument-Bromage H, Tempst P, Moore MA, Massague J. Hematopoiesis controlled by distinct TIF1gamma and Smad4 branches of the TGFbeta pathway. *Cell* **125**, 929-941 (2006).

39. Mali P*, et al.* RNA-guided human genome engineering via Cas9. *Science* **339**, 823-826 (2013).

40. Ran FA, Hsu PD, Wright J, Agarwala V, Scott DA, Zhang F. Genome engineering using the CRISPR-Cas9 system. *Nat Protoc* **8**, 2281-2308 (2013).

41. Sanjana NE, Shalem O, Zhang F. Improved vectors and genome-wide libraries for CRISPR screening. *Nat Methods* **11**, 783-784 (2014).
